# Proteomic Profiling of *Cocos nucifera* L. Zygotic Embryos during Maturation of Dwarf and Tall Cultivars: The Dynamics of Carbohydrate and Fatty Acid Metabolism

**DOI:** 10.3390/ijms25158507

**Published:** 2024-08-04

**Authors:** María Inés Granados-Alegría, Blondy Canto-Canché, Rufino Gómez-Tah, Jean Wildort Félix, Miguel Tzec-Simá, Eliel Ruiz-May, Ignacio Islas-Flores

**Affiliations:** 1 Unidad de Biología Integrativa, Centro de Investigación Científica de Yucatán, A.C., Calle 43 No. 130 x 32 y 34, Colonia Chuburná de Hidalgo, Mérida C.P. 97205, Yucatán, Mexico; granados.alegria@gmail.com (M.I.G.-A.); felixjeandewildort@yahoo.fr (J.W.F.); tzecmyr@cicy.mx (M.T.-S.); 2 Unidad de Biotecnología, Centro de Investigación Científica de Yucatán, A.C., Calle 43 No. 130 x 32 y 34, Colonia Chuburná de Hidalgo, Mérida C.P. 97205, Yucatán, Mexico; cantocanche@cicy.mx (B.C.-C.); rufino26gt@gmail.com (R.G.-T.); 3 Red de Estudios Moleculares Avanzados, Instituto de Ecología, A.C., Carretera Antigua a Coatepec 351, Colonia El Haya, Xalapa C.P. 91073, Veracruz, Mexico

**Keywords:** *Cocos nucifera*, embryo development, zygotic embryo, solid endosperm, TMT proteomics

## Abstract

There is a limited number of studies analyzing the molecular and biochemical processes regulating the metabolism of the maturation of *Cocos nucifera* L. zygotic embryos. Our research focused on the regulation of carbohydrate and lipid metabolic pathways occurring at three developmental stages of embryos from the Mexican Pacific tall (MPT) and the Yucatan green dwarf (YGD) cultivars. We used the TMT-synchronous precursor selection (SPS)-MS3 strategy to analyze the dynamics of proteomes from both embryos; 1044 and 540 proteins were determined for the MPT and YGD, respectively. A comparison of the differentially accumulated proteins (DAPs) revealed that the biological processes (BP) enriched in the MPT embryo included the glyoxylate and dicarboxylate metabolism along with fatty acid degradation, while in YGD, the nitrogen metabolism and pentose phosphate pathway were the most enriched BPs. Findings suggest that the MPT embryos use fatty acids to sustain a higher glycolytic/gluconeogenic metabolism than the YGD embryos. Moreover, the YGD proteome was enriched with proteins associated with biotic or abiotic stresses, e.g., peroxidase and catalase. The goal of this study was to highlight the differences in the regulation of carbohydrate and lipid metabolic pathways during the maturation of coconut YGD and MPT zygotic embryos.

## 1. Introduction

Coconut palm (*Cocos nucifera* L.) is an appreciated crop in the Arecaceae family with 63.7 million tons of palm-fruit-derived products produced worldwide [1,2]. There are two phenotypically recognized coconut varieties classified by their height, robustness, and breeding habits: the dwarf and tall varieties [3,4]. Tall coconuts are 10–15 m taller than dwarf coconuts, which have a maximum height of 8–10 m. Regarding pollination, the palms of tall varieties release their pollen when their feminine flowers are not receptive, thus favoring cross-pollination and genetic variability; in contrast, dwarf varieties release their pollen at times coinciding with receptivity in feminine flowers, resulting in a high percentage of auto-pollination [5].

Six to seven months post-pollination, the ovary becomes a fully grown fruit, measuring 150–200 mm and contains the immature coconut seed. The immature seed is composed of a soft, creamy, brown endocarp, followed by a layer of brown cells termed “testa-cells”, a liquid endosperm known as coconut water [6], and cells forming a jelly-like solid endosperm together with a poorly differentiated zygotic embryo.

Throughout the maturation process of the coconut seed, the tissues and embryo undergo synchronized biochemical and physiological transformations [7,8,9,10]. However, limited information is available in relation to the lipid and carbohydrate metabolism regulation among coconut varieties.

Morpho-histological and biochemical studies based on omics approaches have significantly improved our understanding of gene expression, ovule fertilization, zygotic embryogenesis, and seed and embryo development and maturation [4,11,12,13,14]. Proteomics, as an emerging omics tool, provided an advantage in profiling key protein players with respective post-translational modifications. Additionally, proteomics has fostered the identification of proteins that function as biochemical switches, which coordinate embryo and seed metabolic pathways while they grow and differentiate [15,16]. For example, in oleaginous seeds, Ramzan et al. [17] analyzed the proteome of *Jatropha curcas* zygotic embryos during maturation, finding enrichment of proteins involved in defense and proteolytic activity, as well as in lipid and carbohydrate metabolic pathways. Likewise, Huang et al. [18], using nano-proteomics, found that proteins involved in translation and auxin signaling were crucial for the development of *Arabidopsis thaliana* globular and heart embryos.

Regarding the coconut solid endosperm, a few proteomics studies have profiled its protein composition [19,20]. Recently, Félix et al. [4], using quantitative TMT–SPS–MS3, found dynamic changes in the proteins associated with carbohydrate and lipid metabolic pathways during maturation of the endosperms of Mexican Pacific tall and the Yucatan green dwarf coconut cultivars.

While several proteomics investigations of coconut have focused on solid endosperms, very little has been investigated of the coconut zygotic embryos (ZEs) or somatic embryos (SEs). Lakshmi and Rajesh [21] used MALDI-TOF/TOF-MS to compare the proteomes of various ZE and SE stages and found seven shared proteins between SE and ZE: β-carotene desaturase and adenylate kinase were associated with ZE, while the eukaryotic initiation factor 4A was linked to SE.

The tall and dwarf coconut cultivars have contrasting metabolic pathways in the seed; the former accumulates a greater amount of lipids in the liquid and solid endosperms, while the latter contains a higher amount of carbohydrates [4]. However, it is not clear if these metabolic behaviors are shared with their zygotic embryos. In this study, using a TMT–SPS–MS3 approach, 1044 proteins were identified in the Mexican Pacific tall (MPT) and 540 proteins in the Yucatan green dwarf (YGD) embryos. The comparison of the differentially accumulated proteins (DAPs) between the MPT and YGD embryos highlighted the glyoxylate and dicarboxylate metabolism, fatty acid degradation, and pyruvate metabolism, among other metabolic pathways in the MPT. In the YGD embryo, nitrogen metabolism, pentose phosphate, and purine metabolism were among the most enriched pathways. Independent of the cultivar, in this study, it was found that embryos have a larger proteome related to carbohydrate and fatty acid metabolism compared to solid endosperms [4].

To the best of our knowledge, this is the first study conducted on coconut zygotic embryos, and it reveals differences in the proteomes of MPT and YGD cultivars, as well as in the regulation of lipid and carbohydrate metabolic pathways during coconut embryo maturation.

## 2. Results

### 2.1. Development and Characteristics of Cocos nucifera Embryos

**Figure 1:**
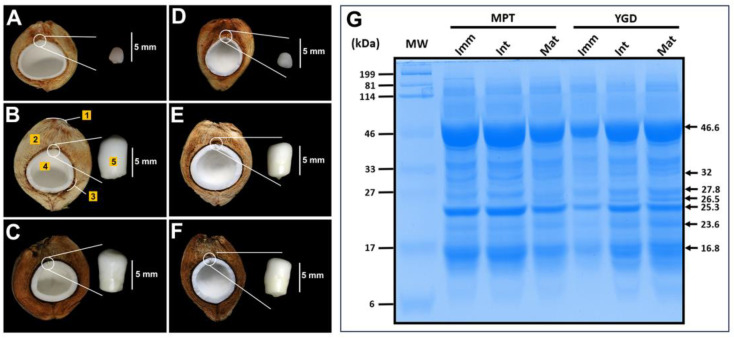
Visual features of coconut embryos during maturation and their protein profiles at different stages of maturity. (**A**–**C**) Seed and embryos of the Mexican Pacific tall (MPT) and (**D**–**F**) the Yucatan green dwarf (YGD) cultivars. (**G**) Protein patterns on a 12% SDS–PAGE of the different stages of development of coconut embryos. Imm = immature, Int = intermediate, Mat = mature, MW = molecular weight, kDa = kilodaltons. In (**B**), 1 = epicarp; 2 = mesocarp; 3 = endocarp; 4 = solid endosperm; 5 = zygotic embryo.

To characterize the morphological and biochemical changes occurring during the maturation of coconut seeds, fruits at increasing stages of maturity were compared. The epicarp of the MPT and the YGD coconut fruit cultivars at the immature and intermediate stages is green and turns brown when mature; the whole process of development and maturation of the fruit takes 12–14 months. The mesocarp at the immature stage is hydrated, with a whitish to slightly brown color, while the solid endosperm is soft and thin (Figure 1A,D). The solid endosperm encloses the zygotic embryo, and during this stage, the embryo is white to creamy in color, soft, conical, and below 5 mm in size. As the fruit progresses to the intermediate stage, the mesocarp becomes less hydrated and more fibrous than the immature stage; the solid endosperm thickens and hardens; and the embryo becomes white–yellowish with a cylindrical shape (Figure 1B,E). At the mature stage, the mesocarp is dehydrated with abundant brown fibers forming the husk, and the solid endosperm is thick and hard. The mature embryo is white–yellowish in color and cylindrical in shape, with a hard and compact consistency (Figure 1C,F).

The SDS–PAGE analysis of soluble protein extracts of zygotic embryos showed polypeptides ranging from 6 to 199 kDa; three polypeptides with molecular weights of 46.6, 25.3, and 16.8 kDa were the most abundant both in MPT and YGD cultivars (Figure 1G). At the protein profile level, only a few differences were observed: two polypeptides with molecular weights of 32 and 27.8 kDa were present only in embryos of immature and intermediate stages of MPT cultivar, while two polypeptides of 26.5 and 23.6 kDa were observed only in the embryo of intermediate and mature stages of the YGD cultivar (Figure 1G).

In summary, the morphological and biochemical results evidenced differences between cultivars and between the different maturity stages.

### 2.2. Identification of the TMT-Labeled Proteins and Functional Classification

**Figure 2:**
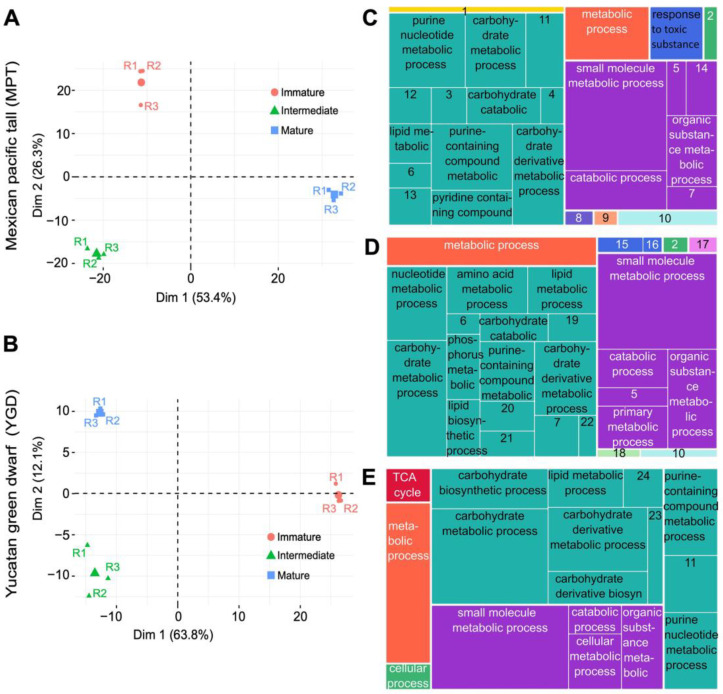
Comparative proteomics analysis of coconut MPT and YGD embryos during the maturation process. Principal component analysis (PCA) for (**A**) MPT and (**B**) YGD; samples were normalized by scaling to a range (0 to 1); R1, R2, and R3 specify the biological replicates in PCA. Treemaps corresponding to enriched biological processes based on (**C**) “global core” proteins, (**D**) MPT-unique proteins, and (**E**) YGD-unique proteins. Each box of the same color represents same biological process, and the larger the box, the greater the enrichment. Note: 1 = cellular aldehyde metabolic process; 2 = cellular process; 3 = energy derivation by oxidation of organic compounds; 4 = ketone catabolic process; 5 = cellular metabolic process; 6 = sulfur compound metabolic process; 7 = organonitrogen compound metabolic process; 8 = response to stimulus; 9 = cell wall organization or biogenesis; 10 = organic hydroxy compound metabolic process; 11 = generation of precursor metabolites and energy; 12 = amino acid metabolic process; 13 = phosphorus metabolic process; 14 = primary metabolic process; 15 = response to toxic substance; 16 = cellular response to phosphate starvation; 17 = cellular metabolic compound salvage; 18 = reactive oxygen species metabolic process; 19 = protein modification by small protein conjugation; 20 = purine-containing compound biosynthetic process; 21 = pyridine-containing compound metabolic process; 22 = organonitrogen compound biosynthetic process; 23 = organic substance catabolic process; 24 = cellular lipid metabolic process.

To quantitatively compare the proteomes of the MPT and YGD coconut embryos and to associate the proteins with their metabolic pathways, proteins from their different maturity stages were TAG-labeled. The TMT–SPS–MS3 approach revealed 1044 proteins in the MPT embryos (Table S1) and 540 proteins in the YGD embryos (Table S2). From these proteins, 656 and 153 were unique (only present in a particular cultivar) for MPT and YGD embryos, respectively, while 388 proteins were shared by both coconut cultivars, thus being the “core” set of proteins. The principal component analysis (PCA) showed that the biological replicates were grouped, supporting the success of the sample classification, collection, and processing; similarly, the embryo proteins were grouped according to coconut cultivar and maturity stages, suggesting that there are distinct proteome profiles for each of the three maturation stages (Figure 2A,B).

The GO-enriched terms of the “core” and the unique proteins in the MPT or YGD embryos were based on *Arabidopsis* protein homologs; the results were summarized and clustered using Revigo and visualized as TreeMaps. The “core” proteome was enriched with proteins involved in the carbohydrate metabolic process, carbohydrate derivative metabolic process, the purine-containing compound metabolic process, lipid metabolic process, and pyridine-containing compound metabolic process (Figure 2C). The enriched unique metabolic processes associated with the MPT embryo were the following: cellular response to phosphate starvation (number 16 in treemap) and reactive oxygen species metabolic process (number 18 in treemap) (Figure 2D). While, in the YGD embryos, the enriched unique metabolic process was tricarboxylic acid cycle (Figure 2E). Thus, the GO enrichment analysis proved that the carbohydrate and lipid metabolic pathways were enriched in coconut embryos during maturation.

**Figure 3:**
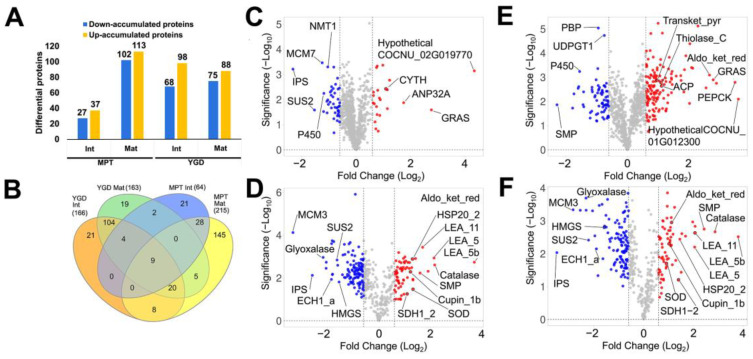
Dynamic behavior of differential proteins during development and maturation of MPT and YGD embryos. (**A**) The number of differentially accumulated proteins identified in the intermediate (Int) and mature (Mat). (**B**) Venn diagram of differential proteins identified in each sample (**C**–**E**). Volcano plots representing the significantly modulated proteins in MPT (**C**,**E**) or YGD (**D**,**F**) cultivars. Up-accumulated proteins are in red; down-accumulated proteins are in blue; non-differentially accumulated proteins are in gray. NMT1 = phosphoethanolamine N-methyltransferase 1, MCM7 = DNA replication licensing factor MCM7, MCM3 = DNA replication licensing factor MCM3, IPS = inositol-3-phosphate synthase, SUS2 = sucrose synthase 2, P450 = cytochrome P450 94C1, CYTH = putative scopoletin glucosyltransferase, ANP32A = acidic leucine-rich nuclear phosphoprotein 32-related protein1, GRAS = scarecrow-like protein 4, LEA_5 = late embryogenesis abundant protein 5, LEA_5b = late embryogenesis abundant protein 5b, LEA_11 = late embryogenesis abundant protein 11 kDa, SMP = seed maturation protein, PEPCK = phosphoenolpyruvate carboxykinase (ATP), Catalase = catalase isozyme, PBP = protein MOTHER of FT and TFL1, Transket_pyr = transketolase chloroplastic, Thiolase_C = 3-ketoacyl-CoA thiolase 2 peroxisomal, Aldo_ket_red = aldo/keto reductase family, Glyoxalase = glyoxalase I family protein, HMGS = hydroxymethylglutaryl-CoA synthase, ECH1_a = enoyl-CoA hydratase isomerase family, HSP20_2 = 16.9 kDa class I heat shock protein 2, Cupin_1b = vicilin-like antimicrobial peptides 2-2, SOD = putative superoxide dismutase [Cu-Zn], SDH1-2 = succinate dehydrogenase 1-2.

To learn more about the regulation of the proteome in the coconut embryos, the number of differentially accumulated proteins (DAPs) was determined with a log2FC value of 0.66 (≤−0.58) for down-accumulated proteins and log2FC of 1.5 (≥0.58) for up-accumulated proteins. Figure 3A shows the number of differentially up- and down-accumulated proteins, while Figure 3B displays the unique and shared proteins between stages and cultivars. Nine proteins were shared in all ratios of both cultivars (Figure 3B). We refer to the ratio Int/Imm as the intermediate stage and the Mat/Imm ratio as the mature stage.

Volcano plots were constructed to illustrate the significant differentially abundant proteins in coconut embryos. In the MPT embryos, the most accumulated proteins in the intermediate and mature stages were hypothetical COCNU_02G019770, scarecrow-like protein, and putative scopoletin glucosyltransferase (Figure 3C,E). The acidic leucine-rich nuclear phosphoprotein 32-related protein 1 was only up-accumulated at the intermediate stage (Figure 3C), while the aldo_keto_reductase, acyl carrier protein 1 chloroplastic, 3-ketoacyl-CoA thiolase 2 peroxisomal, and transketolase chloroplastic, among others, were up-accumulated at the mature stage (Figure 3E).

The proteins in the MPT that showed the greatest down-accumulation in the intermediate stage (Figure 3C) were DNA replication licensing factors MCM7 and MCM3, inositol-3-phosphate synthase, phosphoethanolamine N-methyltransferase 1, sucrose synthase 2, cytochrome P450 94C1, among others. For the mature stage (Figure 3E), the most down-accumulated proteins were seed maturation protein, cytochrome P450 94C1, protein MOTHER of FT and TFL1, and the scopoletin glucosyltransferase-like (UDPGT1).

In the YGD embryos, among the proteins with the largest up-accumulation at the intermediate and mature stage were late embryogenesis abundant protein 5b, protein 5a and 11 kDa, catalase isozyme 2, seed maturation protein, putative superoxide dismutase [Cu-Zn], 16.9 kDa class I heat shock protein 2, vicilin-like antimicrobial peptides 2-2, aldo/keto reductase family, and succinate dehydrogenase 1-2, among others (Figure 3D,F).

The down-accumulated proteins in the YGD embryos in the intermediate and mature stages (Figure 3D,F) were DNA replication licensing factor MCM3, inositol-3-phosphate synthase, glyoxalase 1 family protein, sucrose synthase 2, enoyl–CoA hydratase isomerase, and the hydroxymethylglutaryl–CoA synthase. The analysis of the enriched DAPs supported metabolic differences during the maturation stages of the MPT and YGD embryos, with those of MPT being mainly related to carbohydrate metabolic pathway and the YGD response to biotic and abiotic stresses.

### 2.3. The Enriched GO Terms

**Figure 4:**
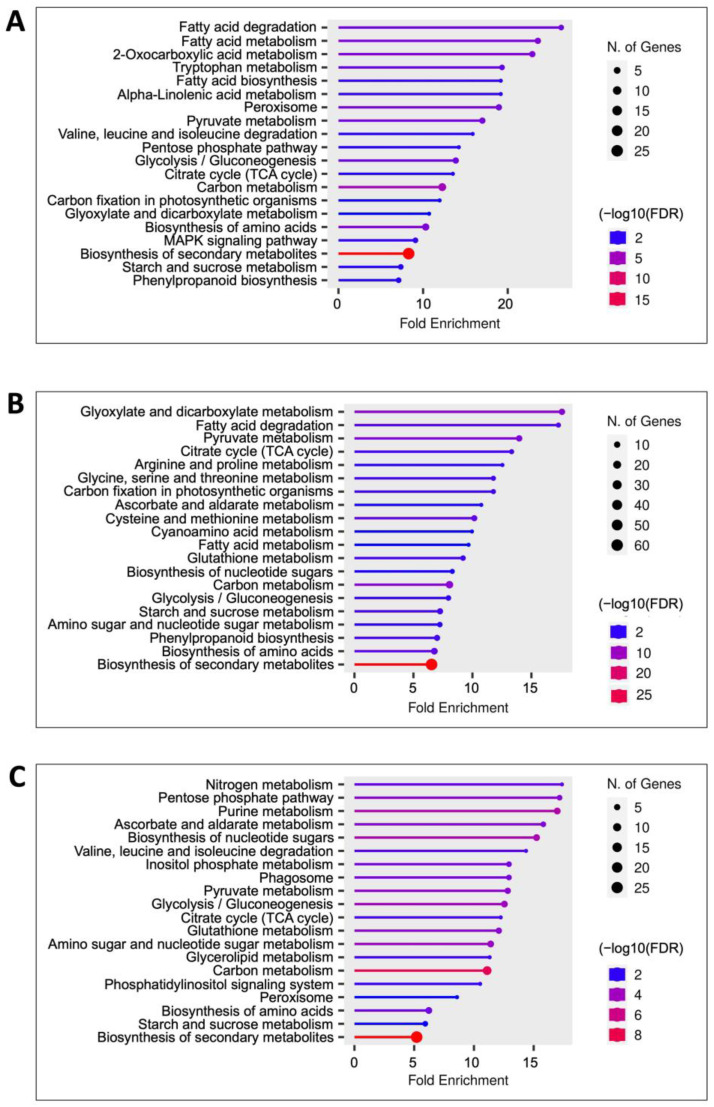
Gene ontology of the biological processes of DAPs identified in the core proteome or unique DAPs for the YGD or MPT embryos. (**A**) Fold enrichment of the core DAPs from YGD and MPT embryos and (**B**) for unique DAPs of MPT or (**C**) YGD embryos. The size of the circles at the end of the line is related to the number of genes. The *X*-axis refers to the fold enrichment and the *Y*-axis to the GO term. The lines are colored by a red–blue gradient with −log10(FDR), blue color means a value of 2 of –log10(FDR) indicating a low significant result, purple and magenta color means a middle significant result and red color means a major significant result.

To determine the fatty acid and carbohydrate metabolic pathways that were associated with the enriched DAPs of the MPT and YGD, a GO term classification based on *Arabidopsis* protein homologs was carried out. The identification of the most enriched GO terms among the DAPs was based on *Arabidopsis* protein homologs. In the identified “core” DAPs, the MPT and YGD shared DAPs, with the highest fold enrichment belonging to the following pathways: fatty acid degradation, fatty acid metabolism, 2-oxo carboxylic acid metabolism, fatty acid biosynthesis, pentose phosphate pathway, and the glyoxylate and dicarboxylate metabolism, among others (Figure 4A).

Biosynthesis of secondary metabolites was the metabolic pathway with the largest number of shared proteins; the MPT and YGD embryos shared 20 proteins in total. However, careful revision of these proteins revealed that only the cytosolic sulfotransferase 16 (SOT16) and 3-epi-6-deoxocathasterone 23-monooxygenase (CYP90D1) were directly involved in glucosinolate or brassinosteroids biosynthesis, respectively (Figure 4A), while the other proteins participate in metabolic pathways which produce potential precursors for lignin, terpenoids, and phenols, such as the shikimate and mevalonate pathways.

Regarding the MPT embryos’ DAPs, the most enriched GO terms (fold enrichment > 8) were glyoxylate and dicarboxylate metabolism, fatty acid degradation pyruvate metabolism and citrate cycle, among others (Figure 4B). As observed in the “core” proteome, the pathway with the higher number of DAPs in MPT embryos was “biosynthesis of secondary metabolites”. The DAPs unique to the MPT embryos were squalene monooxygenase SE1 (SQE1), caffeoyl–CoA O-methyltransferase 1 (CCOAMT), glycerate dehydrogenase, peroxisomal (HPR), hydroxy-delta-5-steroid dehydrogenase, 3-beta- and steroid delta-isomerase 1 (3BETAHSD/D3), glucan-endo-1,3-beta-glucosidase (BGLU20), and dehydroquinate dehydratase (EMB3004), among others. These proteins are different from those found in the core DAPs group, explaining why they are among the unique proteins found in the MPT embryos.

In the case of the YGD embryos (Figure 4C), the pathways with fold enrichment > 10 were nitrogen metabolism, pentose phosphate pathway and purine metabolism, citrate cycle, ascorbate and aldarate metabolism, biosynthesis of nucleotide sugars, valine, leucine and isoleucine degradation, and inositol phosphate metabolism, among others.

As observed in the core DAPs and MPT-unique DAPs, biosynthesis of secondary metabolites was the pathway identified in YGD with a large number of proteins. Some of the 24 proteins identified include the following: delta-aminolevulinic acid dehydratase 2 chloroplastic (HEMB2), 3-hydroxy-3-methylglutaryl-coenzyme A reductase 2 (HMG2), 4-coumarate-CoA ligase 4 (4CL4), hydroxymethylglutaryl-CoA synthase (HMGS), and alpha-amino-adipic semialdehyde synthase (SDH1-1).

Most pathways that were identified as unique to MPT or YGD were in fact present in both cultivars, but the differential accumulation of certain proteins in each coconut cultivar resulted in the pathways being classified as unique for some of the cultivars.

In summary, the DAPs in MPT and YGD embryos are involved in lipid, carbohydrate and amino acid metabolic pathways.

**Figure 5:**
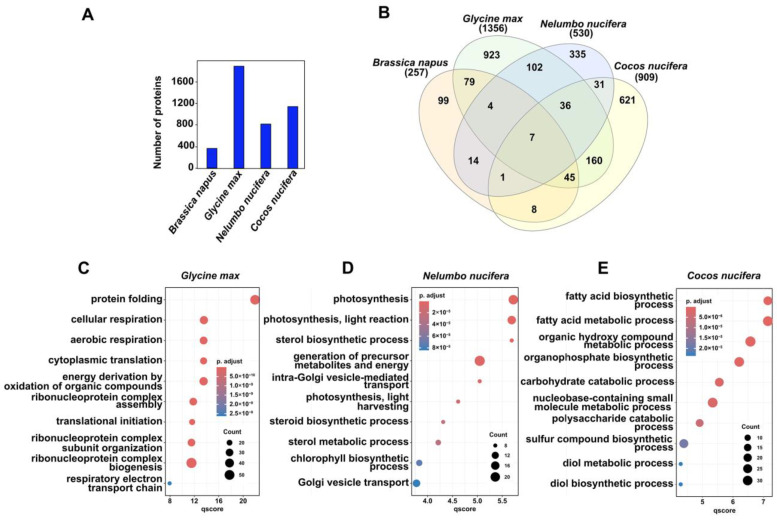
Cross-comparison of the coconut embryo proteome with proteomes reported for oleaginous and non-oleaginous embryos. (**A**) Number of proteins identified in each study; (**B**) Venn diagrams of proteins identified in embryos of *G. max* Wei et al. [22], *B. napus* Lorenz et al. [23], *N. nucifera* Zhang et al. [16], and *C. nucifera* (this study). *Arabidopsis* database, National Center for Biotechnology Information (NCBI), and UniProt databases were used to identify protein homologs. GO enrichment of biological processes related to particular proteins identified in (**C**) *G. max*, (**D**) *N. nucifera*, and (**E**) *C. nucifera* (this study). The size of the dot represents the number of nodes where the contrast qscore intersects with a metabolic pathway. A major count indicates a major number of genes associated with a specific metabolic pathway.

Thus, to explore whether the global coconut embryo proteome shares similarities with the proteomes described during the maturation of other plants, we compared the coconut embryo proteomes with those of oleaginous crops, *Brassica napus* and the *Glycine max* and the non-oleaginous, *Nelumbo nucifera*. Differences in the number of proteins were observed; *G. max* had the most abundant proteome, followed by *C. nucifera*, *N. nucifera*, and *B. napus* (Figure 5A).

All embryos shared a “core” of 7 proteins, while the three oleaginous embryos shared 52 proteins; *C. nucifera* and *G. max* shared 248 proteins, *C. nucifera* and *B. napus* shared 61 proteins, while *C. nucifera* and *N. nucifera* shared 75 proteins (Figure 5B; Table S5). Among the shared proteins of all these plant embryos, there were proteins involved in fatty acid metabolism, i.e., enoyl-(acyl carrier protein) reductase and acyl-acyl-carrier-protein desaturase; lipid metabolism, i.e., diacylglycerol kinase; stress response, i.e., heat shock protein; biosynthesis of vitamin C in plants, i.e., GDP-mannose 3-5-epimerase; acetaldehyde metabolism, i.e., aldehyde dehydrogenase family protein, and oxidoreductase protein associated with flavoproteins, i.e., pyridine nucleotide–disulfide oxidoreductase. Different biological processes were enriched in each plant embryo, even those belonging to the oleaginous group. Protein folding, cellular respiration, aerobic respiration, cytoplasmic translation, energy derivation by oxidation of organic compounds, and ribonucleoprotein complex biogenesis, among others, were the most enriched processes in *G. max* (Figure 5C). In the non-oleaginous *N. nucifera*, photosynthesis, photosynthesis light reaction, sterol biosynthetic process, generation of precursor metabolites and energy, intra-Golgi vesicle-mediated transport, and photosynthesis light harvesting were some of the most enriched processes (Figure 5D); while, in our study, the metabolic processes enriched in the *C. nucifera* embryos were fatty acid biosynthetic process, fatty acid metabolic process, organic hydroxy compound metabolic process, organophosphate biosynthetic process, carbohydrate catabolic process, nucleobase-containing small molecule metabolic process, and polysaccharide catabolic process (Figure 5E). These findings reveal that different metabolic pathways are associated with the embryos of different kinds of seeds.

### 2.4. Carbohydrate- and Lipid-Metabolic Pathways Are the Two Central Pathways in Coconut YGD and MPT Embryos

**Figure 6:**
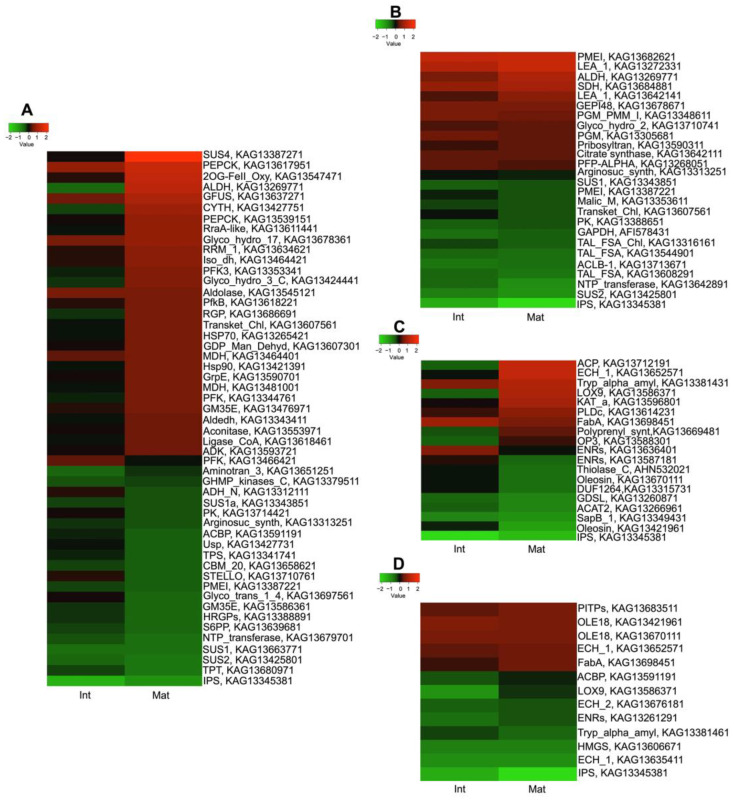
Heatmap of differentially accumulated proteins (DAPs) related to carbohydrate and lipid in zygotic embryos at the three stages of maturation of Yucatan green dwarf and Mexican Pacific tall cultivars. The enzymes related to carbohydrate in (**A**) MPT and (**B**) YGD and the lipids in (**C**) MPT and (**D**) YGD embryos at intermediate and mature stages. The heatmaps were generated using the log2FC values in heatmapper.ca. The color gradient from green to red indicates from a low to high abundance of proteins. The accession number of proteins is on the right side of this Figure. The shorter names on the right side of the heatmaps correspond with PEPCK = phosphoenolpyruvate carboxykinase (ATP), CYTH = putative scopoletin glucosyl-transferase, ALDH = aldehyde dehydrogenase family 7 member A1, PFK3 = ATP-dependent 6-phosphofructokinase 3, 2OG-FeII_Oxy = 2-oxoglutarate-dependent dioxygenase-related family protein, GDP_Man = GDP-mannose 4,6 dehydratase, RraA-like = 4-hydroxy-4-methyl-2-oxoglutarate aldolase, RGP = alpha-1,4-glucan-protein synthase, SUS4 = sucrose synthase 4, Iso_dh = 3-isopropylmalate dehydrogenase 2 chloroplastic, Aldolase = fructose-bisphosphate aldolase 1 chloroplastic, Transket_pyr = Transketolase chloroplastic, ADH_N = Alcohol dehydrogenase class-3, SUS1 = sucrose synthase 1, PK = pyruvate kinase, Argino-suc_synth = argininosuccinate synthase, PMEI = pectinesterase, GM35E = GDP-mannose 3,5-epimerase, HRGPs = Hydroxyproline-rich glycoprotein family protein, S6PP = Sucrose-phosphate synthase, NTP_transferase = Putative Glucose-1-phosphate adenylyltransferase small subunit 1 chloroplastic, SUS1 = sucrose synthase 1, SUS2 = sucrose synthase 2, TPT = Triose phosphate phosphate translocator, IPS = inositol phosphate synthase, ACP = acyl carrier protein, ECH1_b = enoyl-CoA hydratase isomerase family, Tryp_alpha_amyl = non-specific lipid-transfer protein, LOX9 = lipoxygenase 9, KAT_a = 3-ketoacyl-CoA thiolase 2 peroxisomal, PLDc = phospholipase D alpha 1-like protein, FabA = 3-hydroxyacyl-[acyl-carrier-protein] dehydratase FabZ-like, Polyprenyl_synt = geranylgeranyl pyrophosphate synthase large subunit 1 chloroplastic, OP3 = 12-oxophytodienoate reductase, ENRs = Enoyl-(ACP) reductase, Thiolase_C = thiolase, OLE18 = oleosin 18, DUF1264 = oil body-associated protein 1A-like, GDSL = GDSL esterase lipase, ACAT2 = acetyl-CoA acetyltransferase cytosolic 1, SapB_1 = Saposin-like type B, PITPs = phosphatidylglycerol/phosphatidylinositol transfer protein, ACBP = acyl-CoA-binding protein, ECH2 = enoyl-CoA hydratase isomerase family, HMGS = hydroxymethylglutaryl-CoA synthase, ECH1_a = enoyl-CoA hydratase isomerase family, ACLB-1 = citrate synthase beta chain protein 1.

The DAPs accumulation trends related to carbohydrate metabolism were identified for intermediate and mature stages. The log2FC results showed that in the MPT and YGD, there were 272 and 179 total DAPs, respectively (Table S3). In the case of MPT at the intermediate and mature stages, there were 51 proteins related to carbohydrate metabolism (Figure 6A); while, in YGD embryos, there were 26 proteins (Figure 6B). Seven proteins were present in both cultivars, i.e., aldehyde dehydrogenase family 2 member B7 mitochondrial, argininosuccinate synthase, pectinesterase, transketolase chloroplastic (Figure 6A,B); and, among them, the sucrose synthase 1, sucrose synthase 2, and inositol-3-phosphate synthase were some of the proteins most down-accumulated at intermediate and mature stages of the MPT cultivar (Figure 6A).

Sucrose synthase 4 and ATP-dependent 6-phosphofructokinase 3 proteins up-accumulated at the mature stage. Moreover, phosphoenolpyruvate carboxykinase (KAG13617951) and aldehyde dehydrogenase family 2 member B7 mitochondrial were similarly accumulated at the mature stage, being some of the most up-accumulated proteins at that stage (Figure 6A).

In the case of the YGD embryos, the putative glucose-1-phosphate adenylyltransferase small subunit 1 chloroplastic remained down-accumulated at intermediate and mature stages (Figure 6B). The citrate synthase beta chain protein 1 and the transaldolase-like protein were down-accumulated at the intermediate and mature stages. On the other hand, the transketolase chloroplastic protein was stable at the intermediate stage, and then it was down-accumulated at the mature stage (Figure 6B).

Concerning the proteins that were up-accumulated at the intermediate and mature stages in the YGD, the aldehyde dehydrogenase family 2 member B7 mitochondrial, the UDP–glucose 4-epimerase GEPI48 and the putative phosphoglucomutase cytoplasmic 1 were identified (Figure 6B).

A comparison of the carbohydrate proteomes of the MPT and YGD embryos was conducted with the proteomes reported for the solid endosperms of these cultivars (Félix et al., 2023 [4]). It was found that the MPT embryos have a higher number of up-accumulated DAPs at the mature stage compared with solid endosperms of both cultivars, but the log2FC values of the most up-accumulated DAPs were similar in the solid endosperms of both cultivars (Table S4).

On the other hand, the heatmap of the DAPs associated with lipid metabolism showed 19 proteins in the MPT embryos and 13 in YGD embryos (Figure 6C,D). Regarding MPT, the log2FC evidenced that at the intermediate and mature stages, GDSL esterase/lipase LIP-4 and acetyl-CoA acetyltransferase cytosolic 1 (thiolase C) were only slightly down-accumulated (Figure 6C). Regarding the up-accumulated proteins, thiolase_chloroplastic and the enoyl-CoA hydratase isomerase family were up-accumulated at the mature stage, but they did not change at the intermediate stage. In addition, the highest up-accumulated proteins in the MPT zygotic embryos were observed at the mature stage (Figure 6C).

In the YGD, at the intermediate stage, the lipoxygenase 9, the enoyl-CoA hydratase isomerase (ECH1a), and the hydroxymethylglutaryl-CoA synthase were down-accumulated (Figure 6D). Concerning the glyoxysomal fatty acid beta-oxidation multifunctional protein MFP-a, it was slightly up-accumulated at the intermediate stage (Figure 6D). Again, the fold changes of the proteins associated with fatty acid and carbohydrate metabolic pathways support that significant metabolic differences exist between MPT and YGD embryos.

A comparison of the proteomes involved in fatty acid metabolism between the YGD and MPT embryos with their respective solid endosperms would be very informative regarding their metabolic coordination. A higher number of DAPs were identified in embryos (twenty-three); only eight of these were detected in solid endosperms in both cultivars. Although the embryos had a higher number of proteins, the highest log2FC values were observed in the solid endosperms of the MPT and YGD cultivars; these proteins included 3-hydroxyacyl-[acyl-carrier-protein] dehydratase FabZ-like, the acetyl-CoA acetyltransferase cytosolic 1, and the acyl–CoA-binding protein. The first aforementioned protein showed the highest up-accumulation in the MPT cultivar at the intermediate stage (Table S6). Regarding the 3-hydroxyacyl-[acyl-carrier-protein] dehydratase FabZ-like protein, it was gradually up-accumulated in the YGD embryos, while in MPT embryos, it was up-accumulated at the intermediate stage, and then remained the same during maturation (Table S6). In the solid endosperm of YGD, this protein was first down-accumulated at the intermediate stage, while in the MPT, the protein showed gradual up-accumulation (Table S6). The results support the specific coordination of enzymes involved in fatty acid metabolism in the solid endosperm and in the zygotic embryo, with proteins being most up-accumulated in MPT compared to YGD embryos.

### 2.5. Main Carbohydrate and Lipid Metabolic Pathways Identified in Coconut Embryos

**Figure 7:**
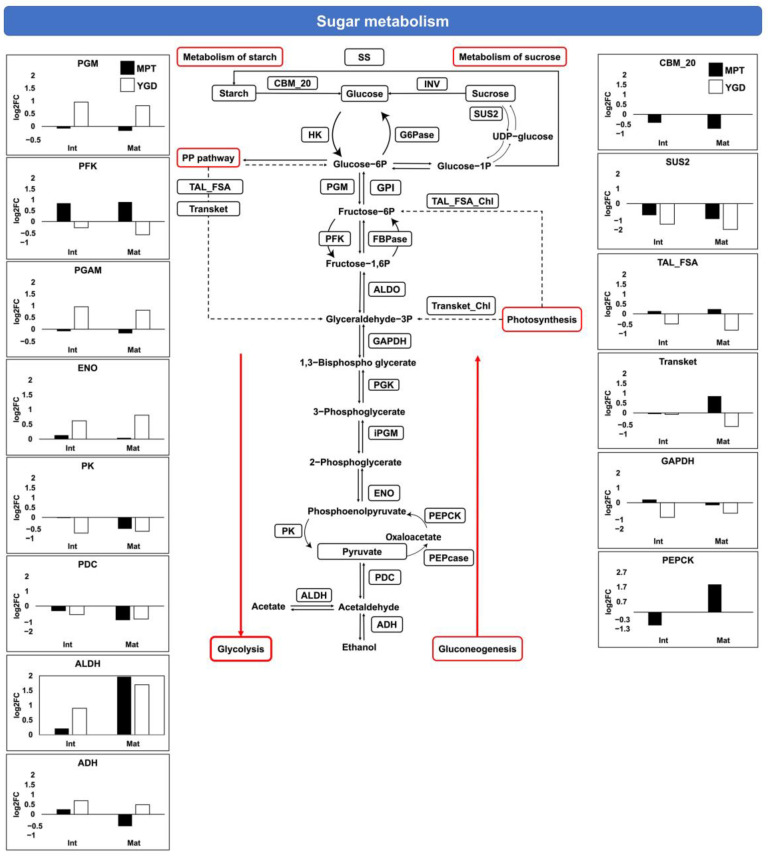
Overview of the glycolysis/gluconeogenesis, pentose phosphate pathway, photosynthesis and sucrose and starch metabolism in zygotic embryos of Yucatan green dwarf and Mexican Pacific tall cultivars. Black bars in the graphs indicate MPT embryos while empty bars correspond to YGD embryos at the intermediate and mature stages. Metabolic pathways are outlined in red. The dotted arrows represent several reaction steps that connect pathways, and the continuous arrow represents a direct reaction of the pathway. PGM = putative phosphoglucomutase cytoplasmic 1 (KAG 13348611); PFK = ATP-dependent 6-phosphofructokinase 3 (KAG13353341 for MPT and KAG13466421 for YGD); PGAM = 2,3-bisphosphoglycerate-independent phosphoglycerate mutase (KAG13655941); ENO = enolase (KAG13269591 for MPT and KAG13652571 for YGD); PK = pyruvate kinase (KAG13388651); PDC = pyvuvate decarboxylase 2 (KAG13380341); ALDH = aldehyde dehydrogenase family 2 member B7 mitochondrial (KAG13269771); ADH = alcohol dehydrogenase 3 (KAG13312111); PEPCK = phosphoenolpyruvate carboxykinase (KAG13539151); GAPDH = glyceraldehyde-3-phosphate dehydrogenase (AYJ721721 for MPT and AFI578431 for YGD); TAL_FSA = transaldolase cytosolic; Transket = transketolase cytosolic; TAL_FSA_Chl = transaldolase fructose-6-phosphate aldolase (KAG13316161); Transket_Chl = transketolase chloroplastic (KAG13607561); SUS2 = sucrose synthase 2 (KAG13425801); CBM_20 = starch binding protein (KAG13658621); FBPase = fructose 1,6-bisphosphatase; INV = invertase; HK = hexokinase; G6Pase = glucose 6-phosphatase; SS = starch synthase; GPI = glucose-6-phosphate isomerase (KAG13480901); ALDO = fructose-bisphosphate aldolase 1 (KAG13633721); PEPcase = phosphoenolpyruvate carboxylase.

**Figure 8:**
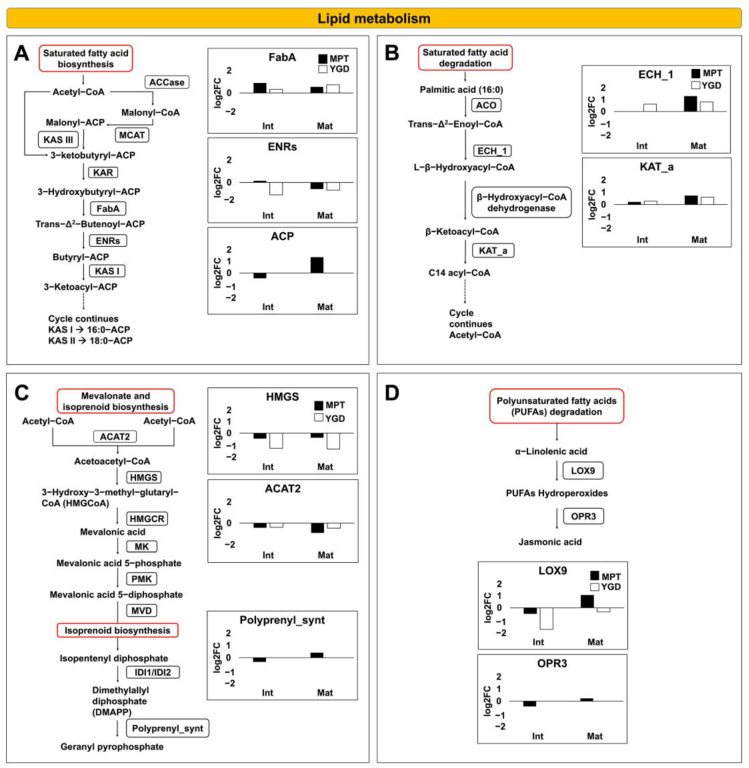
Overview of the saturated and unsaturated fatty acid metabolic pathways and mevalonate and isoprenoid biosynthesis at intermediate and mature stages of YGD and MPT zygotic embryos. (**A**) saturated fatty acid biosynthesis, (**B**) saturated fatty acid degradation, (**C**) mevalonate and isoprenoid biosynthesis, (**D**) polyunsaturated fatty acids (PUFAs) degradation. The black bars correspond to MPT, while the empty bars correspond to YGD. The dotted arrows represent several reaction steps that connect pathways, the continuous arrows represent a direct reaction of the pathway. The boxes outlined in red represent metabolic pathways. ACSL = putative long chain acyl-CoA synthetase; CPT1A = carnitine palmitoyltransferase 1; CPT2 = carnitine palmitoyltransferase 1; ACADVL = acyl-CoA dehydrogenase; echA1 = enoyl-CoA hydratase; HACL1 = 2-hydroxy acyl-CoA lyase; fadA = acetyl-CoA acyltransferase; ACAT = acetyl-CoA acetyltransferase, cytosolic 1; Polyprenyl_synt = geranylgeranyl pyrophosphate synthase large subunit 1 chloroplastic.

Because our results suggest differences in the carbohydrate and lipid metabolic pathways between the embryos of the MPT and YGD cultivars, we analyzed the DAPs using the KEGG database to determine the up- or down-accumulation of proteins involved in glycolysis/gluconeogenesis and lipid metabolism (Figure 7 and Figure 8). Six proteins involved in glycolysis were detected in the zygotic embryos of the YGD and MPT cultivars, while two proteins that function in photosynthesis were present as well (Figure 7). Among the most up-accumulated glycolytic proteins in the MPT cultivar at the mature stage were aldehyde dehydrogenase and phosphofructokinase. In the case of the YGD embryos, aldehyde dehydrogenase and the cytoplasmic phosphoglucomutase were the most up-accumulated at the intermediate and mature stages (Figure 7). With respect to the gluconeogenesis pathway, the most up-accumulated proteins at the mature stage in the MPT embryos were the phosphoenolpyruvate carboxykinase, followed by transketolase chloroplastic.

Regarding photosynthesis and carbon fixation, the transketolase chloroplastic of the MPT embryos showed no change at the intermediate stage and was then up-accumulated at the mature stage. In the YGD, the transketolase chloroplastic was down-accumulated at the mature stage. In the MPT embryos, the transaldolase chloroplastic was stable at the intermediate and mature stages; in the YGD embryos, it remained almost with no change at the intermediate stage, with down-accumulation at the mature stage (Figure 7). Regarding the pentose phosphate pathway, the transaldolase in the MPT embryo showed no changes during the maturation process, while in the YGD embryos, it showed gradual down-accumulation at the intermediate and mature stages (Figure 7). Biosynthesis of sucrose or starch can occur from the hexoses, glucose-6-phosphate and fructose-6-phosphate or trioses, as glyceraldehyde-3-phosphate was produced in the pentose phosphate pathway or photosynthesis [24]. In our analysis, the sucrose synthase 2 in the embryos of both cultivars was gradually down-accumulated at the intermediate stage and mature stages; while the starch binding domain protein CBM_20 was only detected in the MPT embryos, with a gradual down-accumulation during maturation (Figure 7). Together, results show that proteins related to carbohydrate metabolism are more active in the MPT embryos than in the YGD embryos.

To better visualize the fatty acid metabolic pathway during the maturation of the MPT and YGD embryo, the up- and down-accumulation of related proteins were summarized. Of the MPT and YGD embryo, three proteins involved in the biosynthesis of saturated fatty acids were identified. The 3-hydroxyacyl-[ACP] dehydratase FabZ-like (FabA) and the enoyl-(ACP) reductase (ENRs) were present in both embryos, while the chloroplastic acyl carrier protein (ACP) was detected only in the MPT embryos (Figure 8A). The ACP protein was down-accumulated at the intermediate stage and then up-accumulated at the mature stages. The protein FabA followed a gradual up-accumulation at the intermediate and mature in YGD embryo, while in the MPT embryo, it up-accumulated at the intermediate stage and then showed a slight down-accumulation at the mature stage. Regarding the ENRs protein, it showed no change at the intermediate stage in the MPT embryos, while in the YGD embryos, it was down-accumulated at the intermediate and mature stages (Figure 8A).

Regarding the saturated fatty acid degradation, two proteins were identified in the MPT and YGD embryos, the enoyl–CoA hydratase and the 3-ketoacyl–CoA thiolase peroxisomal and both proteins were gradually up-accumulated at the intermediate and mature stages (Figure 8B).

In the case of the mevalonate biosynthetic pathway, hydroxymethylglutaryl–CoA synthase and the acetyl–CoA acetyltransferase cytosolic were the two proteins detected in the MPT and YGD embryos; both proteins showed down-accumulation during maturation (Figure 8C). Furthermore, in the isoprenoid biosynthetic pathway, only the putative heterodimeric geranylgeranyl pyrophosphate synthase large subunit 1 chloroplastic was present in the MPT embryos, but it was not observed in the YGD embryos; this enzyme was down-accumulated at the intermediate stage and remained at the same level at the mature stage (Figure 8C).

In the case of polyunsaturated fatty acids (PUFAs) degradation, lipoxygenase 9 was detected in the YGD and MPT embryos. In MPT, this protein showed down-accumulation at the intermediate stage and then showed a slight up-accumulation at the mature stage, while, in the YGD embryo, this protein was down-accumulated at the intermediate and mature stage (Figure 8D). The 12-oxophytodienoate reductase was detected only in the MPT embryos, with down-accumulation at the intermediate stage, and then up-accumulation at the mature stage (Figure 8D).

In summary, the proteins involved in lipid metabolism were more accumulated during the maturation of the MPT embryos than the YGD embryos.

## 3. Discussion

### 3.1. Characteristics of Coconut Zygotic Embryo

Plant seeds can be classified into two categories according to their nutrient–storage organ: cotyledon-dominant or endosperm-dominant. In the case of the latter, embryos have a single cotyledon (monocot) or cotyledon-like organ (e.g., scutellum storage organ), and the embryo is embedded in the endosperm [25,26].

In the case of the coconut, the zygotic embryo (Figure 1) develops and matures inside the solid endosperm [8]. However, until now, it was unknown whether the embryos coordinate their biochemical metabolism with seed endosperms, thus playing a role in determination of seed fate for a given cultivar or vice versa. In our study, it was observed that maturing embryos grow and change their length and shape actively, growing from less than 5 mm to close to 8 mm and from conical to cylindrical in shape (Figure 1A–F).

The proteins and the roles they play at different stages of zygotic embryo development are integral for correct embryo maturation [27]. Protein dynamics in this study were similar to those described by Islas-Flores et al. [28] in experiments of protein phosphorylation during coconut zygotic embryo development, and also with those described in zygotic embryos of *Pinus pinaster* [29], as well as in the embryo axis of avocado (*Persea americana*) where proteins show a low level of accumulation at immature embryo stages but increased as they reached maturity [13,27].

### 3.2. TMT–SPS–MS3 Analysis in the Coconut Zygotic Embryos

The maturation process of plant zygotic embryos involves multiple metabolic pathways and biological processes that must maintain tight coordination with seed filling, maturation, and control of the metabolic stress employed in embryo cell division, cell expansion, and cell differentiation [30]. The TMT–SPS–MS3 provides a detailed view of the general proteome involved in MPT or YGD coconut zygotic embryo maturation, and our first findings from using this strategy evidenced differences between zygotic embryos from these cultivars since 1044 proteins were found in the MPT (Table S1) and 540 in the YGD embryos (Table S2). Our proteomics results are similar to those obtained for the zygotic embryos of lotus (*N. nucifera*), where, using tandem mass tag-labeled proteomics, 5477 proteins were identified, 815 of them showing changes in abundance at 21, 27, and 40 days of dehydration treatment [16]. To the best of our knowledge, this work is the first of its kind focusing on a TMT–SPS–MS3 quantitative evaluation of the proteomes of different developmental stages of two coconut cultivars.

In the “global core” and unique proteomes revealed during the maturation process of the coconut zygotic embryos of both cultivars, the two largest biological processes associated with maturation were organic substance metabolic process and small molecule metabolic process (Figure 2C–E). Proteomes using or producing organic acids may be involved in the lipid metabolic process and the synthesis of sugars through the gluconeogenic pathway, while proteomes related to small molecule metabolic process may be involved in the synthesis of amino acids and nucleotides, as reported in pro-embryo and embryo development in *B. napus* [31].

The volcano plots showed up- and down-accumulated proteins in both cultivars (Figure 3A,B). Some of the proteins that showed up-accumulation at all stages of maturation in the MPT and YGD embryos were associated with protection against dehydration, heat shock, and oxidative stresses, i.e., LEA1, LEA 5b, heat shock 90–6 proteins, catalase, and glutathione S-transferase. Moreover, scarecrow-like protein (GRAS), a gibberellin transcriptional repressor [32] was also present at all evaluated embryo stages as well (Figure 3C,E). Embryo maturation is a stressful condition that imposes severe metabolic requirements on seed and endosperm cellularization; the process involves maintaining a low concentration of reactive oxygen species as well as controlled regulation of plant hormone concentrations, e.g., abscisic acid in *Arabidopsis thaliana* [30] and gibberellins in coconut solid endosperm and embryo in order to prevent germination before seed maturity [33,34]. Our findings on the presence of a higher number of proteins that detoxify reactive oxygen species in YGD than in MPT embryos suggest that the dwarf coconut is more resistant to stress-inducing ROS [34]. When compared with embryos of other oleaginous plants (i.e., *G. max*, *B. napus*) and the non-oleaginous *N. nucifera*, the 18.1 kDa class I heat shock protein (KAG13306661) was found in the proteome of all these embryos, indicating that it is a common plant embryo protein.

Other proteins involved in overcoming abiotic and biotic stresses, such as glutathione S-transferase DHAR2-like, glutaredoxin, superoxide dismutase [Cu-Zn], catalase isozyme 2, calreticulin, a mannoside lectin possibly involved in the host immune system [35], and the annexin-like protein RJ4 (Table S5), were also identified in the *G. max* and *B. napus* oleaginous embryos [36]. Among the oleaginous embryos, we visualized a lactoylglutathione lyase (Table S5), a critical enzyme in the detoxification of methylglyoxal, whose elimination is necessary for survival under stress conditions [37,38]. This enzyme was also described in the proteome of the solid endosperm [4] and the metabolome from the liquid endosperm [39] of *C. nucifera*, probably useful in facing the stress imposed by a highly active glycolysis [4]. Consistently, other proteins shared between *C. nucifera* and *N. nucifera* were also associated with the plants’ stress response. Protein DJ-1 is an oxidation/reduction sensor, anti-oxidant, and molecular chaperone which deglycosylates methylglyoxal- and glyoxal-proteins releasing the repaired proteins [40,41]. Other stress-related embryo proteins included a 1-cys peroxiredoxin, which regulates peroxide-induced oxidation and activation of a stress-activated MAP kinase and H_2_O_2_ reduction in the presence of thioredoxin [42,43]; a selenoprotein H, which is a redox-sensing, DNA-binding protein that up-regulates genes involved in glutathione synthesis [44]; and a sulfite oxidase, an essential enzyme that oxidizes harmful sulfite to sulfate. All these proteins indicate that plant embryos face and mitigate different stresses, including metabolic, abiotic, and biotic stresses, and this is consistent with metabolic scenario uncovered here in the proteomes of the coconut embryos.

### 3.3. DAPs Related to Amino Acid Metabolism and Biosynthesis of Secondary Metabolites

In this study, it was determined that in the “core” DAPs, amino acid metabolism was enriched, i.e., tryptophan metabolism and valine, leucine, and isoleucine degradation (Figure 4A). Taken altogether, the results support a tight and essential link among the biological processes involving fatty acid, carbohydrate, and amino acid metabolic pathways in coconut zygotic embryo, independently of the cultivar. In the oleaginous crop, *P. americana*, amino acids were mostly accumulated in the zygotic embryo, while the enzymes associated with their biosynthesis were mostly present in the seed testa cells [13]. Amino acids are essential for zygotic embryo maturation in the oil producers, *Thlaspi arvense* and *J. curcas* [45,46]. Similarly, the zygotic embryo of *Arabidopsis* is the final acceptor of maternal translocated hormones, peptides, and nutrients which guarantee embryo growth [47,48]. In coconut embryos, this is probably similar; valine, leucine, and isoleucine degradation and tryptophan metabolism were enriched in both cultivars (Figure 4A), and, in addition, glycine, serine, and threonine metabolism were enriched in the MPT (Figure 4B). Altogether, these results support the key role of amino acid metabolism during coconut zygotic embryo development.

As previously mentioned, the biosynthesis of secondary metabolites and phenylpropanoid metabolism were also detected as enriched in the “core” DAPs. This evidences that the coconut embryo, independently of the cultivar, contains proteins (e.g., scopoletin glucosyl transferase and cytosolic sulfotransferase 16) that are involved in the biosynthesis of products, such as scopoletin, a coumarin used as chemo-preventive compound [49]; the presence of coumarins and other secondary metabolites demonstrates that coconuts are a viable source of bioactive compounds [50].

The protein scopoletin glucosyl transferase (CYTH) was present at all maturation stages of both cultivars, suggesting the accumulation of scopoletin, which in plants has been associated with the response to plant pathogens [51]. Engineered scopoletin accumulation reduces oxidative stress and disease susceptibility in *Nicotiana benthamiana*, *N. tabacum*, *G. max,* and *Arabidopsis thaliana* plants [52]. This coumarin has been identified in many plant families and in different plant parts, such as the fruit, shoot, root, aerial parts, and flowers [53]. Its presence in plant embryos has been less discussed; in the coconut embryo, it probably participates in the reduction in oxidative stress or as a pre-formed metabolite which helps defend the embryo from pathogens.

### 3.4. Proteins Related to Carbohydrate Metabolism

The most enriched processes in plant embryos are carbohydrate and lipid metabolic pathways [54,55], and the proteome of *C. nucifera* embryos is no different (Figure 3, Figure 4 and Figure 5).

The TCA cycle and pyruvate metabolism were the most representative pathways associated with carbohydrate in MPT (Figure 4B), while, in the YGD zygotic embryos, the most representative pathways were pentose phosphate pathway, pyruvate metabolism, glycolysis/gluconeogenesis, and carbon metabolism (Figure 4C). These metabolic pathways are usually the main avenues in plants for the production or consumption of bioenergy [16,56]. In the embryos of oleaginous *P. americana* and *J. curcas*, glycolysis hydrolyzes carbohydrates to obtain NADH and pyruvate, while the PPP and TCA cycle are the major pathways for the generation of reducing power and precursors for fatty acids [13,17]. The TCA cycle is key in the balance of the cell redox potential during plant embryo development and germination by regulating the levels of malate and citrate present [57]. In addition, a proteomic study carried out on 18-day-post-pollination embryos from the cross of *C. morifolium* × tetraploid *C. nankingense* showed that TCA disbalance resulted in embryo malformation and abortion [58].

Regarding the DAPs related to carbohydrate metabolism, one of the most up-accumulated enzymes at the mature stage in both the YGD and MPT was the aldehyde dehydrogenase family 2 member B7 mitochondrial (Figure 6A,B; Table S4). The aldehyde dehydrogenase protein has been reported as a regulator of plant embryo development and seed viability in *Arabidopsis* and rice [59]. It is likely that in the coconut zygotic embryo, the enzyme dynamically impacts the metabolism of storage products such as starch and sucrose by catalyzing the oxidation of aromatic and aliphatic aldehydes to their corresponding carboxylic acids, thus producing NADH and NADPH [60].

The down-accumulation of the transketolase protein in the YGD zygotic embryos (Figure 6A) suggests that they depend on heterotrophic nutrition; enough amounts of NADH and NADPH arise from the catabolism of carbohydrates [61] in the embryo or in surrounding tissues [4]. On the contrary, in the MPT embryos, the transketolase goes from a basal level at the intermediate stage to being up-accumulated at the mature stage. This suggests that in MPT, the carbohydrate metabolism through PPP and glycolysis is demanded for embryo nutrition; the adjacent solid endosperm contains lower amounts of carbohydrate than in the YGD cultivar [4].

#### Glycolysis/Gluconeogenesis

The DAPs enzymes involved in the glycolysis/gluconeogenesis, i.e., phosphoenolpyruvate carboxykinase (PEPCK) and the phosphofructokinase (PFK), were up-accumulated during the last stage of maturation in the MPT (Figure 7). In *A. thaliana*, PFK and wrinkled (WRI1) transcription factor play a role in the regulation of carbon skeleton channeling to lipid biosynthesis in the embryo [62], which is consistent with higher lipid accumulation in MPT compared to YGD coconuts.

Regarding the glycolysis/gluconeogenesis-related proteins in the YGD embryo, only the phosphoglucomutase (PGM) was up-accumulated during maturation, while glyceraldehyde 3-phosphate dehydrogenase (GAPDH), phosphoglycerate mutase (PGAM), and pyruvate kinase (PK) were down-accumulated (Figure 7). PGM catalyzes the reversible reaction from glucose 1-phosphate to glucose 6-phosphate, but it is also involved in the metabolism of starch during embryo maturation [63].

The up-accumulation of PGM and down-accumulation of GAPDH and PK suggest that the glycolysis pathway is less active in YGD than in MPT. Glucose 1-phosphate from the catabolism of starch or sucrose might be isomerized to glucose 6-phosphate and channeled to PP pathway, which was found highly enriched in MPT (Figure 7). In summary, these results suggest that the MPT zygotic embryo has active glycolysis, while, in the YGD zygotic embryo, the glycolysis pathway is inhibited, perhaps because carbohydrates are readily available in the solid and liquid endosperms of the YGD coconut [4,39].

### 3.5. Proteins Related to Lipid Metabolism

Although coconut is an oleaginous plant, and many reports have characterized its fatty acid content, very little is known about the regulation of the lipid metabolic pathway in coconut. Here, in both YGD and MPT zygotic embryos, the pathway with the highest fold enrichment related to lipid metabolism was fatty acid degradation (Figure 4A and Figure 5E). In YGD, most of the proteins were down-accumulated, and only the glyoxysomal enoyl CoA hydratase was up-accumulated (Figure 8B). The up-accumulation of that enzyme coincides with results seen in the *J. curcas* germinating embryo; stored lipids were degraded through β-oxidation and glyoxylate cycle to provide carbon and energy for embryo development, germination, and seedling growth [17]. The fact that four proteins involved in lipid metabolism were down-accumulated at different stages in the YGD embryo (Figure 8A,C,D) is consistent with low lipid accumulation in YGD seeds. In contrast, in the MPT zygotic embryos, seven proteins of the lipid metabolism pathway were up-accumulated at the mature stage (Figure 8A–D), while only three proteins were down-accumulated at that stage (Figure 8A,C). This finding is congruent with high lipid accumulation in the MPT coconut and shows coordinated lipid regulation between the zygotic embryo and endosperms in coconut.

Glyoxysomal enoyl CoA hydratase and the acetyl–CoA acetyltransferase, cytosolic 1 (ACAT), are two β-oxidation enzymes present among the DAPs in the embryos of both cultivars. ACAT is the predominant protein in germinating seeds of *Arabidopsis* [64], but here, ACAT was down-accumulated in the embryos of both coconut cultivars. This possibly indicates temporal regulation of ACAT in embryos; before germination, it is down-accumulated to preserve acetyl–CoA and energy which could be used during germination.

The findings of enriched metabolic pathway classifications of the unique DAPs in the MPT or YGD embryos were congruent with reports of their respective solid endosperms; the lipid metabolic pathway was enriched in the MPT embryo, while the carbohydrate metabolic pathway was enriched in the YGD embryo, which suggests coordinated regulation in each case, i.e., solid endosperm and embryo. Core DAPs such as LEA, catalase, and isocitrate dehydrogenase were shared proteins found in the embryos of other oleaginous plants and are related to desiccation and stress resistance, probably fundamental processes in plant embryos.

In summary, our data show that in coconut embryos, accurate coordination among fundamental metabolic pathways, i.e., sucrose synthesis, starch metabolism, and fatty biosynthesis, among others, is required for embryo and seed maturation.

## 4. Materials and Methods

### 4.1. Plant Material

The coconut fruits (*C. nucifera* L.) from the Yucatan green dwarf (YGD) and Mexican Pacific tall (MPT) cultivars were collected in San Crisanto, Yucatan, Mexico, at latitude 21°16′11″ north and longitude 88°44′22″ west. The coconut embryos were collected using a maturity-based classification of the fruits: the immature stage consisted of 6–8 month-old fruits, the intermediate stage consisted of 9–10 month-old fruits, and the mature stage consisted of 11–14 month-old fruits [4,39,65]; the length and width of the embryos were determined with a digital Vernier. The embryos were pooled according to their maturity stage and immediately frozen in liquid nitrogen and stored at –80 °C until further analysis. Each maturity point was prepared with three biological replicates.

### 4.2. Embryo Protein Extraction and Quantification

Proteins were extracted using the method described in Félix et al. [4] with some modifications. The frozen embryos were ground in sterile Eppendorf tubes using a 1 mL blunt-sealed tip in the presence of liquid nitrogen until a fine powder was obtained. The powder was homogenized in 250 µL of extraction buffer (50 mM Tris-HCl, pH 7.4 with added 50 mM NaCl, 10% glycerol, 1 mM EDTA, 1 mM EGTA, 5 mM β-mercaptoethanol, 1 mM PMSF, 1/2 of pill of complete^TM^ protease inhibitor cocktail [Roche, Burgess Hill, West Sussex, UK]) and then centrifuged at 13,000× *g* at 4 °C for 10 min. The supernatants were transferred into new Eppendorf tubes and centrifuged again at 13,800× *g* for 15 min at 4 °C; supernatants were recovered and placed in new sterile tubes. The protein concentration was determined according to the Bradford method [66].

### 4.3. Gel Electrophoresis

The total soluble protein (30 µg per sample) was independently loaded onto 12% sodium dodecyl sulfate–polyacrylamide gel electrophoresis (SDS–PAGE), according to the Laemmli protocol [67]. Electrophoresis was performed using the experimental conditions described in Félix et al. [4].

### 4.4. Protein Preparation for TMT Labeling

A thousand (1000) μg of protein was precipitated using the chloroform–methanol method [68,69]. An aliquot of 100 μg of proteins was reduced with 10 mM Tris (2-carboxyethyl) phosphine (TCEP), alkylated with 30 mM iodoacetamide (IA), quenched with 30 mM dithiothreitol (DTT), and precipitated again with cold acetone overnight at −20 °C and centrifuged at 10,000× *g* for 15 min at 4 °C. Pellets were prepared according to Félix et al. [4]; samples were digested overnight with trypsin in a ratio of 1:30 *w*/*w* and stopped at 80 °C [70]. Trypsin-digested peptides were desalted using ziptip tips and dried at 25 °C.

TMT labeling was carried out as described by Félix et al. [4]. Briefly, acetonitrile was added to tandem mass tags (TMT–9plex reagent; Thermo-Fisher, Waltham, MA, USA) and the mixture was gently shaken. According to the manufacturer, the peptide samples were suspended in a 100 mM triethylammonium bicarbonate (TEAB) buffer for five minutes and then conjugated with TMT for one hour at 25 °C. The peptide samples of the embryos of YGD or MPT cultivars were labeled and analyzed separately.

The reagents 126, 127N, and 127C were used to label the peptides of the immature stage embryos; 128C, 129N, 129C for the peptides of the intermediate stage; and 130N, 130C, 131 for the peptides of the mature stage. Peptide labeling was finished after 15 min of incubation at room temperature by adding 5% of hydroxylamine. High-pH reverse-phase C18 cartridges were used to pool and fractionate all samples belonging to the same cultivar. The pooled samples were desalted and cleaned using C18 cartridges and then dried with a CentriVap; Labconco, Kansas City, MO, USA.

### 4.5. Nano LC–MS/MS

The desiccated TMT-labeled embryo peptide samples were dissolved in 0.1% formic acid in LC–MS-grade water and then analyzed as described in Félix et al. [4].

### 4.6. TMT Synchronous Precursor Selection (SPS)-MS3

We used the Orbitrap Fusion Tribid mass spectrometer (Thermo-Fisher Scientific, San Jose, CA, USA) to execute full MS scans with the following settings: maximum injection time of 50 ms, dynamic exclusion 1 at 70 s, resolution of 120,000 (FWHM), scan range of 350–1500 *m*/*z*, AGC of 2.0 × 10^5^, and mass tolerance of 10 ppm. The 20 most prevalent MS1s were separated, and their charge states were adjusted to 27 for the MS2 study. The fragmentation parameters comprised an isobaric tag loss TMT, an in-trap detection ion, a precursor selection mass range of 400–1200 *m*/*z*, a maximum injection time of 50 ms, an AGC of 1.0 × 10^4^, a CID with 35% collision energy and activation Q of 0.25, and MS3 spectra acquired using a 10-notch SPS isolation. Using a maximum injection duration of 120 ms and one microscan, the Orbitrap was used to evaluate the fragmented MS3 precursors at a resolution of 60,000 and a scan range of 120–500 *m*/*z*. Additionally, a 2 *m*/*z* isolation window and 1.0 × 10^5^ AGC were also included.

Proteome Discoverer 2.4 (Thermo-Fisher Scientific Inc.) was used to examine the spectra obtained using MS/MS and (SPS)-MS3. The search engines MS Amanda 2.0 and Mascot server (version 2.4.1, Matrix Science, Boston, MA, USA) and the Sequest HT method were used to process the data.

The *Arabidopsis* family sequences that were retrieved from the PlantCyc (https://plantcyc.org, accesed on 10 July 2023) database were the search targets. Two missed cleavages, carbamidomethylation of cysteine (+57.021 Da) and TMT 9-plex N-terminal/lysine residues (+229.163 Da) were taken into consideration along with full-tryptic protease specificity. Additionally, the dynamic changes in methionine oxidation (+15.995 Da) and deamidation in asparagine/glutamine (+0.984 Da) were taken into consideration with limits of ±10 ppm and ±0.6 Da; protein identification was conducted in the linear ion trap at a lower resolution. The Percolator algorithm was used to filter peptide hits at a maximum of 1% false discovery rate (FDR) [71]. Additionally, the precursor co-isolation filter was adjusted to 45% in the Orbitrap analyzer for TMT measurement at the MS3 level.

### 4.7. Bioinformatic Analysis

Proteome Discoverer 2.4 was used to generate protein and peptide abundance. The number of total peptides was used as the basis for normalization. Protein abundances were used to calculate the fold change (FC) for the stages “Intermediate versus Immature” (Int/Imm) and “Mature versus Immature” (Mat/Imm). The differential proteins were determined using empirical moderated Bayesian statistics with a fold change ≥ 1.5 (log2FC ≥ 0.58) and ≤0.66 (log2FC ≤ −0.58), and PCA-based normalized protein abundances were also computed with the limma package in the R environment version 2024.04.2+764. For the gene ontology enrichment, we used an *Arabidopsis* database, considering ≥80% identity. We used both the ShinyGO 0.76 platform (http://bioinformatics.sdstate.edu/go76/ (accessed on 12 October 2023)) and Cluster Profiler package [72] in the R environment version 2024.04.2+764. We summarized redundant GO terms with the REVIGO software version 1.8.1 (http://revigo.irb.hr, accessed on 12 October 2023). A Manhattan distance was computed as a criterion for ranking hits in the VolcaNoseR platform (https://huygens.science.uva.nl/VolcaNoseR2/, accessed on 15 October 2023). For the volcano plots on the x-axis the log2FC of the intermediate (Int/Imm) and mature (Mat/Imm) stages were used and significant values (*p*-value 0.05) were on the y-axis. The Venn diagram was created with the online tool https://www.interactivenn.net/ (accessed on 15 October 2023) [73].

## 5. Conclusions

Gene ontology analysis of the DAPs from the YGD and MPT embryo proteomes grouped the metabolic pathways into three major groups: proteins related to carbohydrate metabolism, lipid metabolism, and amino acid metabolism. The PP pathway, glycolysis, fatty acid degradation, and valine and leucine degradation were prominent in the YGD embryos. While, for the MPT embryos, the TCA cycle and serine and threonine metabolism were additional to those found in the YGD.

Differences in the regulation of carbohydrate and lipid metabolic pathways during maturation of YGD and MPT zygotic embryos were observed. Glycolysis was less active in the YGD embryos, while the MPT embryos exhibited active glycolysis and fatty acid metabolism. Proteomics evidence suggest that coconut embryos face metabolic, abiotic, and biotic stresses; it is likely that coconut embryos have pre-formed metabolites, such as aldarate and scopoletin, which help mitigate the various stresses faced.

The comparison of the proteins identified in the coconut zygotic embryos to those of *Glycine max* and *Nelumbo nucifera* revealed great metabolic differences among them, with the fatty acid biosynthetic process being the most enriched in coconut, protein folding in *Glycine max*, and photosynthesis and light reactions in *Nelumbo nucifera*. These findings reveal that different metabolic pathways are relevant to the embryos of different kinds of seeds.

Overall, this research provided valuable insights into the complex proteomic processes governing coconut zygotic embryo development. It may be worthwhile to perform coconut embryo metabolomics to validate these results.

The findings of this study are a step forward in the elucidation of the complete proteome of the *Cocos nucifera* embryo, which can be used as the basis for future comparisons with other oilseeds.

## Data Availability

The original contributions presented in the study are included in the article/Supplementary Materials, further inquiries can be directed to the corresponding authors.

## Supplementary Materials

The following supporting information can be downloaded at: https://www.mdpi.com/article/10.3390/ijms25158507/s1.
