# Supplementary material for: Proteomic Profiling of Cocos nucifera L. Zygotic Embryos during Maturation of Dwarf and Tall Cultivars: The Dynamics of Carbohydrate and Fatty Acid Metabolism
Source: Int J Mol Sci. 2024 Aug 4;25(15):8507. doi: 10.3390/ijms25158507 (PMC11312736; doi:10.3390/ijms25158507)
Supplement: Supplementary file 1 [file ijms-25-08507-s001.zip › Legends for the supplementary material_IIF.pdf]

Supplementary material

Table S1. Identities of the total protein sequences obtained from embryos of the Mexican Pacific tall cultivar at three different developmental stages of maturity and the IDs of their protein homologues in the *Arabidopsis thaliana* database at PlantCyc. CV%= coefficient of variation, SD=Standard deviation, T.Test = Student t test.

Table S2. Identities of the total protein sequences obtained from embryos of the Yucatan green dwarf cultivar at three different developmental stages of maturity and the IDs of their protein homologues in the *Arabidopsis thaliana* database at PlantCyc. CV%= coefficient of variation, SD=Standard deviation, T.Test = Student t test.

Table S3. Differential accumulated proteins (DAPs) in embryos of the coconut Mexican Pacific tall (MPT) and Yucatan green dwarf (YGD) cultivars and their protein homologs in the *Arabidopsis thaliana* database. N/A= Non-applicable

Table S4. Comparison of the differential accumulated proteins (DAPs) involved in carbohydrate metabolism in YGD and MPT zygotic embryos and their solid endosperms at three different maturity stages. N/A= Non-applicable

Table S5. Comparison of the proteomes shared among the embryos from *Cocos nucifera*, *Brassica napus*, *Glycine max* and *Nelumbo nucifera*.

Table S6. Comparison of the differential accumulated proteins (DAPs) involved in lipid metabolism in YGD and MPT zygotic embryos and their solid endosperms at three different maturity stages. N/A= Non-applicable
